# A Bayesian spatio-temporal analysis of mortality rates in Spain: application to the COVID-19 2020 outbreak

**DOI:** 10.1186/s12963-021-00259-y

**Published:** 2021-05-31

**Authors:** Pedro Saavedra, Angelo Santana, Luis Bello, José-Miguel Pacheco, Esther Sanjuán

**Affiliations:** 1 Department of Mathematics, University of Las Palmas de Gran Canaria, Las Palmas, Spain; 2 Department of Physical Education and Biomedical and Health Research Universitary Institute, University of Las Palmas de Gran Canaria, Las Palmas, Spain; 3 Department of Animal Pathology and Production, Bromatology and Food Technology, University of Las Palmas de Gran Canaria, Las Palmas, Spain

**Keywords:** COVID-19, Excess of deaths, Spatio-temporal models, Standardized mortality ratios, Integrated nested Laplace approximation

## Abstract

**Background:**

The number of deaths attributable to COVID-19 in Spain has been highly controversial since it is problematic to tell apart deaths having COVID as the main cause from those provoked by the aggravation by the viral infection of other underlying health problems. In addition, overburdening of health system led to an increase in mortality due to the scarcity of adequate medical care, at the same time confinement measures could have contributed to the decrease in mortality from certain causes. Our aim is to compare the number of deaths observed in 2020 with the projection for the same period obtained from a sequence of previous years. Thus, this computed mortality excess could be considered as the real impact of the COVID-19 on the mortality rates.

**Methods:**

The population was split into four age groups, namely: (< 50; 50–64; 65–74; 75 and over). For each one, a projection of the death numbers for the year 2020, based on the interval 2008–2020, was estimated using a Bayesian spatio-temporal model. In each one, spatial, sex, and year effects were included. In addition, a specific effect of the year 2020 was added ("outbreak"). Finally, the excess deaths in year 2020 were estimated as the count of observed deaths minus those projected.

**Results:**

The projected death number for 2020 was 426,970 people, the actual count being 499,104; thus, the total excess of deaths was 72,134. However, this increase was very unequally distributed over the Spanish regions.

**Conclusion:**

Bayesian spatio-temporal models have proved to be a useful tool for estimating the impact of COVID-19 on mortality in Spain in 2020, making it possible to assess how the disease has affected different age groups accounting for effects of sex, spatial variation between regions and time trend over the last few years.

## Background

Data on mortality attributed to coronavirus disease 2019 (COVID-19) in Spain—one of the most affected western European countries—have been very controversial. Disagreements between data-providing sources arise from the difficulty of telling apart patients who died “from COVID-19” as the main cause from those suffering other underlying health problems that together with the virus caused death, i.e., “having contracted COVID-19.” In addition to that, the highly decentralized Spanish Health System has been a serious handicap for a sound data collection, thus leading Spanish health authorities to reconsider the published data: even today, criteria adopted for this count are being discussed. Uncertainties in the approach to the correct diagnosis about excess mortality produced by COVID-19 have also been suggested in other places such as England and Wales [1].

The real impact of the epidemic goes far beyond determining how many people died from COVID-19 or suffered COVID-19. In fact, as indicated in [2], there are people who have died indirectly from COVID (for example, because they had not received adequate medical attention for other problems) and should be considered as side effects of the epidemic. There are also people who actually died from COVID-19, especially in the case of the elderly or seriously ill, but perhaps the disease only accelerated the moment of death, which would have happened anyway. In addition, the lockdown measures that were taken in the country during the first wave of the pandemic also had the effect of reducing mortality from other causes: there were fewer deaths in road accidents; it is even possible that isolation facilitated the reduction of infections due to influenza viruses, also reducing mortality from this cause. Therefore, any assessment of the impact of COVID on mortality in Spain during 2020 will include a balance between all these effects since it is not possible to estimate them separately.

An attractive way to assess the impact of the epidemic on mortality is to compare the deaths observed in 2020 with those projected from a sequence of previous years. This has been understood by the authors of [3, 4], who have evaluated the total excess of deaths during periods of the epidemic in Italy, by geographical areas, sex, and age, with respect to what was expected from 2015 to 2019 [3], or from previous months [4].

These estimates have sometimes focused on identifying or predicting the trend in mortality rates during a given period of the pandemic ([5] during lockdown in Spain [6]; during a fortnight in India [7]; for a quarter in Italy, Spain, and France [8]; for a 4-month period in Iran). Other authors have focused on the investigation of the relationship of spatial patterns with say ecological factors in the USA [9].

The application of spatio-temporal study methods has proven to be ideal to optimize the observation of epidemics behavior, using an information exchange based on the influence of location proximity and time closeness [10]. These methods have been deemed essential to describe the spread and to make decisions about the mitigation of the pandemic [11, 12].

The purpose of this study is to compare the mortality rates observed in the year 2020 with the trend projected for this year based on the sequence of mortality rates in years 2008–2020 according to *age group*, *sex*, and *autonomous communities* (administratively, Spain is divided into seventeen autonomous communities). Obviously, mortality rates over time cannot be assumed to be generated by a stationary process, which would imply that rates obtained from averages of death counts in past years (e.g., 2015–2019, using a 5-year period as is usually done in mortality studies) do not estimate any characteristic parameters of the process. Therefore, this assumption does not offer an optimal way to make a projection for the year 2020. Since mortality patterns showed differences between age groups, a spatio-temporal model for the death counts was estimated for each group. In each one the effects of sex, autonomous community and year (sequence 2008–2020) were included. In addition, a specific effect for year 2020 ("outbreak") was added. Models were estimated in the Bayesian framework via the *integrated nested Laplace approximation* (INLA). Once the projected deaths for 2020 were estimated, the excess mortality was obtained as the difference between observed and projected deaths.

## Methods

### Data source

All data have been obtained from the Spanish official Institute of National Statistics (INE). Population data can be found in [13], mortality data by autonomous community, sex, and age until 2019 have been downloaded from [14], and mortality data for 2020 are located in [15].

For each one of the *age cohorts* (< 50, 50–64, 65–74, and 75 over), the dataset has the form:

$$ \left\{\left({Pop}_{s,a,t};{Death}_{s,a,t}\right):s= male, female;a=1,\dots, 17;t=2008,\dots, 2020\right\} $$

where *Pop*_*s*, *a*, *t*_ *and Death*_*s*, *a*, *t*_ denote the population size and the number of deaths during 2020, respectively in the cohorts determined by sex (*s*), autonomous community (*a*), and year (*t*). Values of (*a*) correspond to the INE codes of communities.

### Observed mortality rates by sex, community, and time

The mortality rate (deaths by 100,000 persons-year) in cohort {*s, a, t*} was estimated as

$$ {R}_{s,a,t}={10}^5\times \frac{Death_{s,a,t}}{Pop_{s,a,t}} $$

### Expected number of deaths assuming uniformity of the rates by autonomous communities, years, and sex

Under the hypothesis of uniformity of the rates, the expected number of deaths is defined as

$$ {E}_{s\operatorname{}a\operatorname{}t}=r\bullet {Pop}_{s\operatorname{}a\operatorname{}t} $$

where *r* = ∑_*s*, *a*, *t*_*Death*_*s*, *a*, *t*_/∑_*s*, *a*, *t*_*Pop*_*s*, *a*, *t*_ (*total proportion of deaths*)

### Spatio-temporal model to project mortality from 2008–2019 to 2020

In each of the four age groups, we assume that the number of deaths in the {*s*, *a*, *t*} cohort is a random variable with *negative binomial probability distribution* with mean *λ*_*s*, *a*, *t*_ where:

$$ \log \left({\lambda}_{s,a,t}\right)=\alpha +{\beta}_{s,a}+{u}_a+{v}_a+{\gamma}_t+{d}_a\cdot t+{f}_a\cdot {I}_t(2020)+\log \left({E}_{s,a,t}\right) $$

Here, *α* is the intercept, *β*_*s*, *a*_ denotes the interaction *sex-autonomous community* (for all *a* = 1, …, 17, *β*_*female*,*a*_ = *β*_0_ and *β*_*male*,*a*_ = *β*_*a*_), *u*_*a*_ the spatially structured community effect modeling spatial adjacency, *v*_*a*_ the community-specific (unstructured) effect, *γ*_*t*_ the temporally structured effect and *d*_*a*_ a random slope that modulates the trend in each autonomous community. In addition, *f*_*a*_ · *I*_*t*_(2020) models the specific effect of COVID-19 on the excess mortality in 2020 (*outbreak*), being *I*_*t*_(2020) = 1 for *t* = 2020 and zero for *t* < 2020, and *f*_*a*_, the different effects of the epidemic for the autonomous community *a*.

### Estimation

Model parameters were estimated using the Bayesian framework. Briefly, Bayesian methods assume a certain a priori knowledge of the parameters, which is formulated as a probability distribution (prior distribution). This distribution, together with the available data, leads to the so-called posterior distribution, whose characteristic values constitute the parameter estimates. So, the 2.5th and 97.5th percentiles of the posterior distributions yield a credibility interval (CI) for the corresponding parameter. Therefore, the estimation of each parameter of the model is presented via the mean, median, 2.5th and 97.5th percentiles of its posterior probability distribution.

For the four spatio-temporal models, one for each age cohort, we use the following prior distributions, namely: for *α*, we use a centered Gaussian distribution with variance 10^4^ (*α*~*N*(0; 10^4^)), whereas for the sex-autonomous community interactions *β*_*s*, *a*_ we assume priors centered, independent and normally distributed random variables *IIDN*(0; 10^4^).

The spatially structured components **u** = {*u*_*a*_ : *a* = 1, …, 17} were modeled via the corresponding adjacency matrix of the autonomous communities (*h*_*i*, *j*_ = 1 or 0 according on whether the communities *i* and *j* are neighboring or not) by conditional autoregressive priors as was first proposed in [16, 17]. More concretely, we assume that:

$$ {u}_a\mid {\mathbf{u}}_{-a}\sim N\left(\frac{1}{{\mathcal{N}}_a}{\sum}_j{h}_{a,j}{u}_j,{\sigma}_u^2/{\mathcal{N}}_a\right) $$

Here, **u**_−*a*_ = **u** − {*u*_*a*_} and ${\mathcal{N}}_a$ is the number of neighboring communities to {*a*}. For the precisions $1/{\sigma}_v^2,$ a prior *logGamma*(1; 10^5^) was considered. The unstructured *v*_*a*_ was modeled as a centered, independent, and normally distributed random variable; i.e., $IIDN\left(0,{\sigma}_v^2\right)$, being $1/{\sigma}_v^2\sim logGamma\left(1;{10}^5\right)$. The time effect was modeled dynamically as a random walk of order 2, i.e., ${\gamma}_t\left|{\gamma}_{t-1},{\gamma}_{t-2}\sim N\left(2{\gamma}_{t-1}+{\gamma}_{t-2};{\sigma}_{\gamma}^2\right)\right.$ [18]. For $1/{\sigma}_{\gamma}^2$, we consider a prior *logGamma*(1; 10^5^). Finally, for the random effects *d*_*a*_ and *f*_*a*_, we modeled the priors as *IIDN*(0, 10^5^).

The probability distribution of the deaths count was based on the *deviance information criterion* [19]. It is a generalization of the Akaike information criterion (AIC), developed for the comparison of two models in the Bayesian framework. According to this criterion, a value of DIC is associated to each model. Then, given two models, the one with the lower DIC will be preferred.

From this model, the projection of deaths for 2020 in the cohort {*s*, *a*}, based on the sequence 2008–2019 is

$$ {Project}_{s,a}={E}_{s,a,t=2020}\bullet \exp \left(\alpha +{\beta}_{s,a}\cdotp s+{u}_a+{v}_a+{\gamma}_{2020}+{d}_a\cdotp 2020\right) $$

And the rate-ratio between the real rate and project for the (*a*)-community is

$$ {RR}_a=\exp \left({f}_a\right) $$

All models were estimated using the procedure known as integrated nested Laplace approximation (INLA), through the R-interface [20, 21]. Data were analyzed using the R language and environment, version 3.6.1 [22].

## Results

**Fig. 1:**
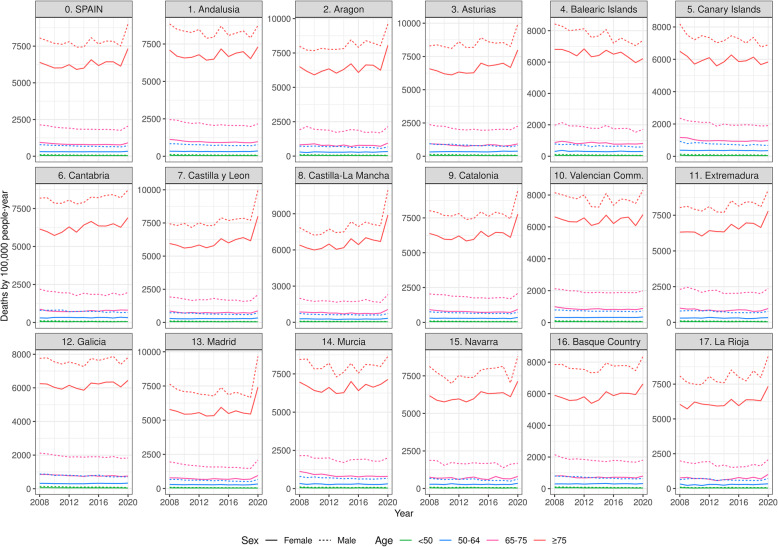
Deaths by 100,000 people-year according to autonomous community, sex, and year

**Fig. 2:**
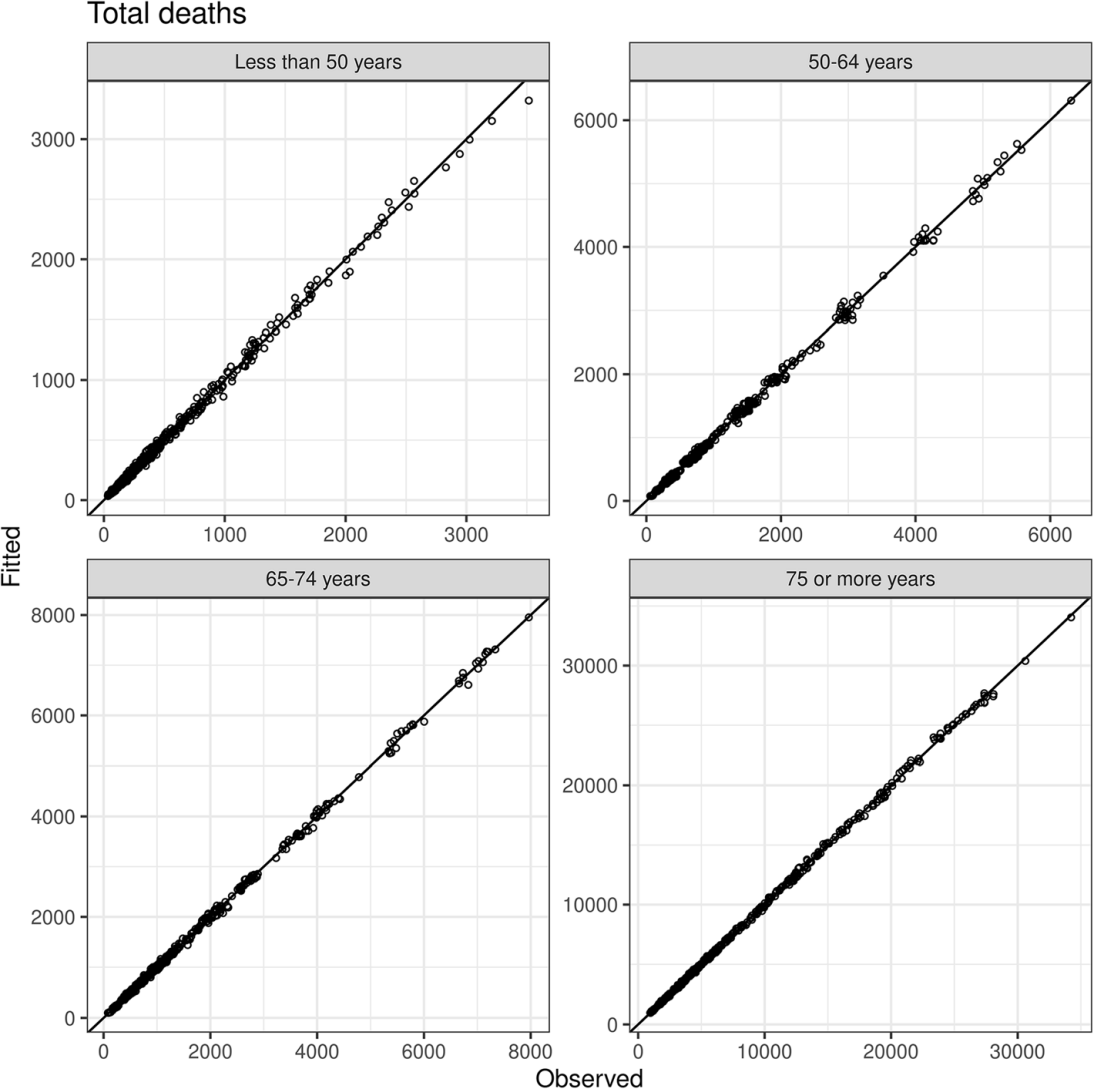
Goodness of fit for the four spatio-temporal models

**Fig. 3:**
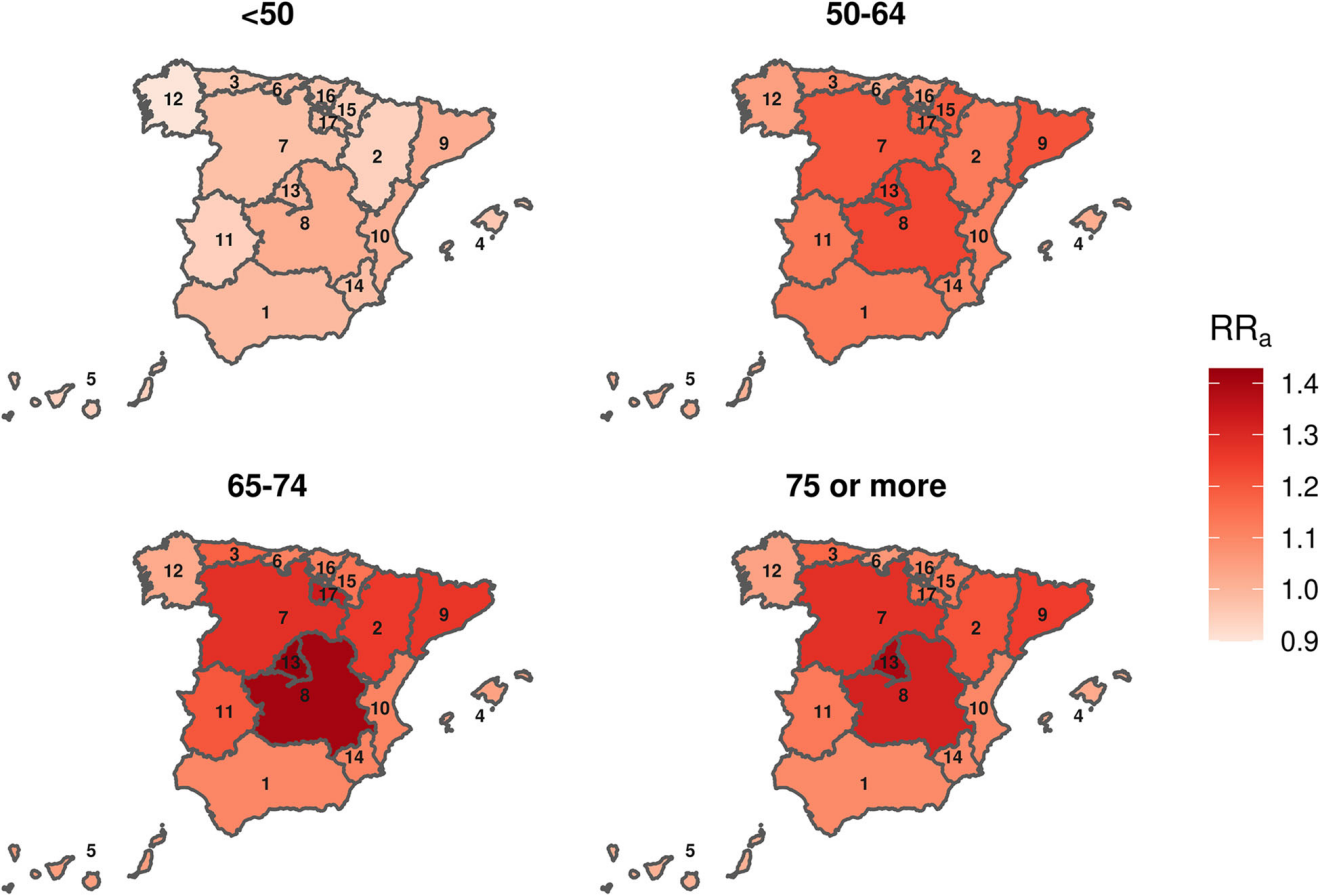
Ratio rates (RR) between observed and projected mortality rates for 2020 in each autonomous community. Projected mortality rates were obtained from the mortality data corresponding to years 2008–2019. Autonomous communities have been numbered as in Table 3

Figure 1 shows the annual evolution of the mortality rates observed from 2008 to 2020 for entire Spain and per Autonomous Community, according to age group and sex. Possible probability distributions for the death counts are the Poisson and negative binomial distributions. According to the deviance information criterion (see Table 1), the negative binomial distribution led to better fits than the Poisson distribution for the models corresponding to the age groups 50–64 years and 75 and over. For the other age groups, both distributions led to similar fits. Figure 2 displays the fitted versus observed death counts for each of the four models, when using the negative binomial distribution. As can be seen, all models present a very good fit. Table 2 shows the observed and projected counts of deaths according to age group. The total number of deaths in the 17 Spanish autonomous communities during 2020 was 499,104, while the number projected by the spatio-temporal models for the same period was 426,970, the difference being 72,134 deaths. However, this increase was very unequally distributed among the communities. Table 3 shows the fitted mortality rates for 2020 (projected and actual) and the mortality rates ratios (real/projected) are also displayed in Fig. 3. In the group of age less than 50 years, there was no significant increase in mortality rates. In the other age groups, Madrid was the community that experienced the highest growth rates (24.8%, 42.8%, and 39.4%) in the groups of 50–64 years, 65–64 years, and 75 and over, respectively). Increases in mortality rates were also high in the two communities adjacent to Madrid (Castilla-La Mancha and Castilla y León) and decreased in all directions but to the northeast. Catalonia also showed growth rates greater than 20% in groups aged 50 and over. In the island communities (the Canary and the Balearic Islands), the mortality rates estimated for 2020 did not show significant increases in relation to those projected in any of the age groups. The relationship between the mortality rates between the sex groups also showed spatial differences. For example, in the Basque Country and in the group aged 75 and over, the mortality rate was 31% higher in men than in women, while in Murcia it was only 20.9% higher.

**Table 1:** Deviance information criterion: DIC values according to model and probability distribution

Probability distribution for the deaths count	< 50 years	50–64 years	65–74 years	75 and over
Poisson	4126.7	4651.9	4547.8	5622.0
Negative binomial	4114.1	4562.6	4556.5	5537.8

**Table 2:** Estimation of the excess of deaths

	***Deaths***		
	Observed	Projected^a^	Excess^b^
< 50 years	15,543	15,817	− 274
50–64 years	48,191	42,498	5693
65–74 years	67,230	56,608	10,622
75 or more years	368,140	312,047	56,093
	499,104	426,970	72,134

^a^Spatio-temporal models: means of the posterior probability distribution

^b^Observed minus projected

**Table 3:** Projected and fitted real mortality rates in 2020 (death by 100,000 people-year) and the corresponding rate ratios (real/projected)

	A: Rate projection for 2020		B: Fitted real rates for 2020		
Autonomous	Females	Males	Females	Males	Rate ratios (B/A)
**Community**	***Age group < 50 years***				
1. Andalusia	41 (39; 43)	77 (74; 81)	41 (39; 43)	77 (73; 81)	0.989 (0.929; 1.054)
2. Aragón	38 (35; 40)	69 (64; 73)	35 (32; 38)	64 (59; 70)	0.942 (0.851; 1.020)
3. Asturias	46 (43; 49)	88 (82; 93)	44 (40; 48)	84 (77; 92)	0.965 (0.879; 1.047)
4. Balearic Islands	37 (34; 39)	68 (64; 72)	35 (32; 38)	65 (60; 71)	0.962 (0.877; 1.042)
5. Canary Islands	43 (40; 45)	79 (75; 84)	40 (37; 43)	75 (69; 80)	0.942 (0.864; 1.014)
6. Cantabria	39 (36; 43)	70 (65; 75)	39 (35; 43)	69 (62; 76)	0.983 (0.895; 1.081)
7. Castilla y León	40 (38; 43)	70 (66; 74)	39 (37; 42)	68 (64; 73)	0.976 (0.902; 1.052)
8. Castilla-La Mancha	36 (34; 38)	66 (62; 70)	37 (34; 40)	67 (63; 73)	1.017 (0.945; 1.111)
9. Catalonia	38 (36; 40)	67 (64; 71)	38 (36; 41)	68 (65; 72)	1.015 (0.954; 1.088)
10. Valencian Comm.	41 (39; 43)	75 (71; 79)	41 (39; 44)	76 (71; 80)	1.006 (0.943; 1.080)
11. Extremadura	38 (35; 41)	73 (69; 78)	36 (32; 39)	69 (63; 75)	0.943 (0.850; 1.023)
12. Galicia	44 (41; 47)	87 (82; 92)	39 (36; 43)	78 (72; 84)	0.899 (0.812; 0.982)
13. Madrid	34 (32; 35)	56 (53; 59)	33 (32; 35)	56 (53; 59)	0.998 (0.935; 1.070)
14. Murcia	39 (36; 41)	71 (67; 76)	38 (35; 41)	70 (65; 75)	0.980 (0.903; 1.061)
15. Navarra	34 (31; 37)	61 (57; 66)	32 (29; 36)	58 (52; 64)	0.954 (0.854; 1.040)
16. Basque Country	40 (38; 43)	70 (66; 74)	39 (36; 42)	68 (63; 73)	0.969 (0.893; 1.045)
17. La Rioja	37 (33; 40)	65 (59; 71)	36 (32; 41)	64 (57; 71)	0.982 (0.887; 1.088)
	***Age group from 50 to 64 years***				
1. Andalusia	301 (290; 314)	706 (679; 734)	342 (325; 360)	800 (760; 841)	1.132 (1.068; 1.200)
2. Aragón	274 (261; 287)	620 (594; 647)	309 (288; 331)	699 (653; 748)	1.128 (1.047; 1.215)
3. Asturias	322 (308; 338)	768 (736; 804)	357 (333; 383)	850 (794; 910)	1.105 (1.026; 1.190)
4. Balearic Islands	288 (272; 303)	617 (587; 646)	293 (270; 316)	627 (582; 675)	1.018 (0.938; 1.102)
5. Canary Islands	335 (320; 350)	703 (673; 732)	338 (317; 360)	709 (667; 754)	1.009 (0.942; 1.080)
6. Cantabria	296 (280; 313)	683 (651; 717)	305 (278; 333)	704 (646; 765)	1.030 (0.940; 1.124)
7. Castilla y León	267 (256; 279)	603 (579; 628)	321 (303; 342)	724 (683; 768)	1.203 (1.126; 1.286)
8. Castilla-La Mancha	255 (244; 268)	590 (566; 617)	315 (295; 336)	727 (684; 775)	1.234 (1.151; 1.325)
9. Catalonia	275 (265; 286)	628 (605; 653)	333 (316; 351)	759 (721; 799)	1.208 (1.139; 1.282)
10. Valencian Comm.	299 (287; 313)	681 (653; 712)	333 (316; 352)	758 (719; 800)	1.113 (1.044; 1.184)
11. Extremadura	281 (267; 295)	686 (655; 716)	318 (296; 342)	776 (724; 832)	1.132 (1.050; 1.222)
12. Galicia	293 (281; 306)	727 (699; 758)	307 (289; 326)	762 (718; 808)	1.048 (0.981; 1.119)
13. Madrid	258 (248; 268)	541 (520; 563)	322 (305; 340)	675 (641; 712)	1.248 (1.174; 1.327)
14. Murcia	270 (257; 284)	628 (600; 656)	299 (278; 321)	695 (649; 744)	1.107 (1.027; 1.193)
15. Navarra	261 (246; 276)	542 (515; 570)	311 (285; 340)	647 (594; 704)	1.193 (1.092; 1.308)
16. Basque Country	286 (274; 299)	645 (619; 671)	304 (285; 323)	684 (644; 728)	1.061 (0.991; 1.135)
17. La Rioja	268 (250; 287)	594 (561; 629)	317 (284; 353)	702 (635; 778)	1.182 (1.067; 1.316)
	***Age group from 65 to 74 years***				
1. Andalusia	880 (847; 911)	1969 (1898; 2038)	970 (942; 1000)	2172 (2110; 2237)	1.102 (1.056; 1.151)
2. Aragón	727 (695; 760)	1720 (1650; 1792)	916 (870; 965)	2166 (2062; 2274)	1.259 (1.185; 1.338)
3. Asturias	772 (738; 807)	1927 (1848; 2007)	913 (866; 961)	2278 (2169; 2392)	1.181 (1.112; 1.255)
4. Balearic Islands	763 (727; 800)	1680 (1607; 1753)	794 (746; 844)	1748 (1648; 1853)	1.041 (0.972; 1.115)
5. Canary Islands	902 (865; 939)	1845 (1773; 1918)	948 (906; 991)	1938 (1855; 2024)	1.050 (0.994; 1.110)
6. Cantabria	719 (682; 759)	1791 (1709; 1878)	803 (746; 863)	1999 (1866; 2137)	1.116 (1.032; 1.206)
7. Castilla y León	691 (664; 720)	1615 (1554; 1679)	884 (848; 920)	2065 (1986; 2146)	1.280 (1.215; 1.347)
8. Castilla-La Mancha	725 (695; 757)	1653 (1588; 1722)	1020 (975; 1067)	2325 (2227; 2427)	1.408 (1.331; 1.489)
9. Catalonia	711 (685; 737)	1677 (1618; 1736)	901 (874; 930)	2125 (2063; 2190)	1.268 (1.214; 1.325)
10. Valencian Comm.	796 (766; 826)	1804 (1738; 1869)	882 (853; 913)	1999 (1934; 2065)	1.108 (1.058; 1.160)
11. Extremadura	790 (755; 827)	1989 (1907; 2073)	950 (898; 1004)	2391 (2269; 2518)	1.201 (1.128; 1.280)
12. Galicia	732 (704; 761)	1802 (1735; 1870)	750 (721; 781)	1846 (1776; 1919)	1.025 (0.974; 1.078)
13. Madrid	650 (626; 674)	1484 (1430; 1539)	928 (899; 959)	2119 (2054; 2187)	1.428 (1.365; 1.495)
14. Murcia	784 (748; 820)	1752 (1676; 1826)	869 (822; 918)	1942 (1842; 2045)	1.108 (1.040; 1.182)
15. Navarra	651 (616; 687)	1544 (1472; 1619)	743 (688; 801)	1763 (1641; 1891)	1.142 (1.054; 1.238)
16. Basque Country	672 (645; 700)	1670 (1606; 1734)	753 (720; 787)	1869 (1792; 1950)	1.120 (1.062; 1.182)
17. La Rioja	674 (632; 719)	1590 (1506; 1680)	900 (818; 990)	2124 (1943; 2319)	1.335 (1.211; 1.473)
	***Age group of 75 or more years***				
1. Andalusia	6594 (5898; 7480)	8101 (7245; 9189)	7218 (7070; 7369)	8860 (8677; 9047)	1.093 (0.963; 1.223)
2. Aragón	6476 (5790; 7349)	8135 (7273; 9231)	7859 (7659; 8063)	9862 (9609; 10122)	1.212 (1.067; 1.359)
3. Asturias	6750 (6034; 7660)	8652 (7733; 9819)	7878 (7672; 8090)	10093 (9825; 10367)	1.166 (1.026; 1.307)
4. Balearic Islands	6080 (5434; 6901)	7229 (6460; 8206)	6232 (6035; 6435)	7410 (7173; 7654)	1.025 (0.901; 1.150)
5. Canary Islands	5707 (5103; 6475)	6957 (6220; 7893)	5763 (5608; 5923)	7022 (6832; 7218)	1.010 (0.889; 1.131)
6. Cantabria	6407 (5724; 7274)	8326 (7438; 9454)	6824 (6593; 7062)	8865 (8562; 9178)	1.065 (0.935; 1.196)
7. Castilla y León	6219 (5561; 7056)	7851 (7020; 8908)	7943 (7768; 8122)	10024 (9802; 10251)	1.280 (1.127; 1.433)
8. Castilla-La Mancha	6801 (6081; 7716)	8217 (7347; 9323)	8961 (8751; 9176)	10831 (10576; 11092)	1.319 (1.161; 1.478)
9. Catalonia	6154 (5505; 6980)	7658 (6850; 8686)	7694 (7538; 7854)	9570 (9374; 9770)	1.250 (1.101; 1.399)
10. Valencian Comm.	6204 (5550; 7038)	7499 (6708; 8507)	6823 (6676; 6973)	8250 (8072; 8432)	1.099 (0.968; 1.230)
11. Extremadura	6753 (6037; 7663)	8359 (7472; 9486)	7644 (7435; 7858)	9456 (9195; 9724)	1.131 (0.995; 1.268)
12. Galicia	6137 (5490; 6961)	7578 (6778; 8595)	6395 (6251; 6542)	7892 (7714; 8076)	1.042 (0.918; 1.166)
13. Madrid	5380 (4812; 6103)	6827 (6106; 7745)	7493 (7337; 7652)	9516 (9317; 9719)	1.394 (1.227; 1.561)
14. Murcia	6521 (5830; 7400)	7882 (7046; 8945)	7151 (6950; 7357)	8643 (8399; 8895)	1.096 (0.964; 1.229)
15. Navarra	6207 (5544; 7047)	7766 (6937; 8819)	7088 (6853; 7330)	8868 (8570; 9174)	1.142 (1.003; 1.283)
16. Basque Country	5873 (5252; 6662)	7700 (6886; 8735)	6511 (6357; 6670)	8530 (8326; 8740)	1.109 (0.977; 1.242)
17. La Rioja	6290 (5610; 7149)	8101 (7226; 9209)	7343 (7042; 7655)	9458 (9067; 9863)	1.167 (1.023; 1.316)

**Fig. 4:**
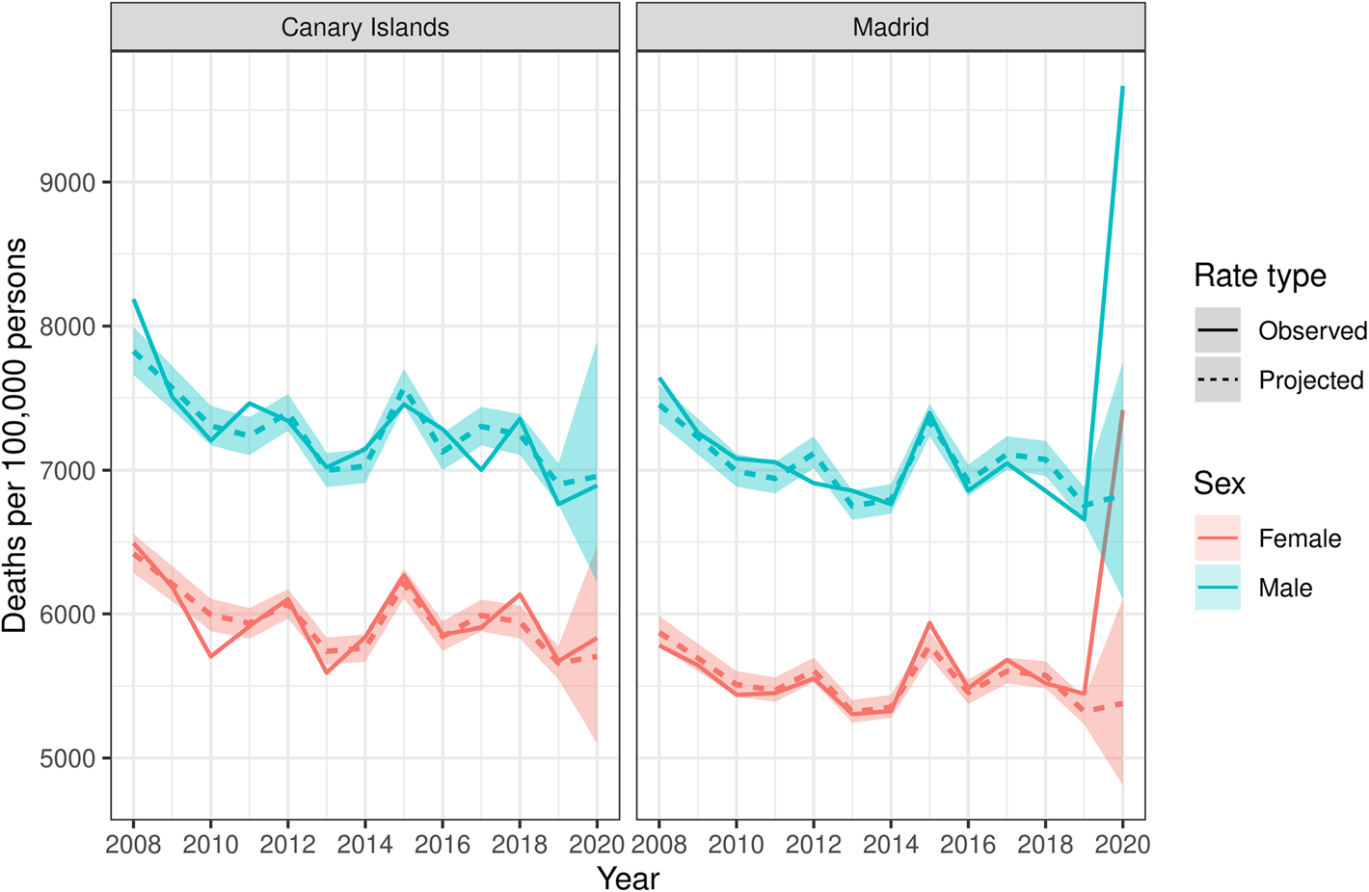
Evolution of mortality rates for the age group of 75 year or over in Canary Islands and Madrid: observed and fitted rates according sex. The temporally effect was modeled dynamically by means of a random walk of order 2. Bands corresponding to the 95% credibility intervals

Figure 4 displays the evolution of the mortality rates for Canary Islands and Madrid for the age group 75 and over. These autonomous communities exhibited the greatest differences in the evolution pattern of mortality rates. The increase in the mortality rate in 2020 was not statistically significant in the Canary Islands (*RR*_*a*_ = 1.01; 95% CI = 0.89; 1.13), while Madrid had the highest in Spain (*RR*_*a*_ = 1.39; 95% CI = 1.23; 1.56).

## Discussion

This study aims to assess the spatial distribution of excess mortality in Spain in 2020 in relation to the projection of mortality for that year based on the 2008–2020 sequence. For this purpose, four spatio-temporal models for the evolution of mortality rates through the 2008–2020 period, including the effects of autonomous community and sex—and the appropriate interactions—were estimated. The impact of COVID-19 in 2020 as the joint result of all effects converging on mortality—as cited in [2]—was evaluated by introducing a specific effect for 2020 (community-year interaction). The modeling of the temporal effect in two components must be highlighted, namely: a general component (*γ*_*t*_) dynamically modeled by means of a random walk of order two plus a specific linear trend for each community (*d*_*a*_). Through these slopes, a significant decreasing trend was detected in mortality rates for the Canary Islands (*d*_*Canary*_ = − 0.0077; 95% CI = − 0.0113; − 0.0042) and an increasing trend for Castilla La Mancha (*d*_*La Mancha*_ = 0.0078; 95% CI = 0.0045; 0.0112). It should be noted that a closer study of the model revealed that the effects of sex were not uniform for all the autonomous communities, and hence a sex-community interaction was introduced through the parameters *β*_*s*, *a*_. Therefore, it is reasonable to think that the model is an adequate tool for estimating the spatial effects of an epidemic during a given period.

Our spatio-temporal model is estimated in the Bayesian framework by using the INLA procedure. INLA does not require iterative computations to obtain an approximation to posterior distributions, thus making it computationally efficient. Alternatively, Markov chain Monte Carlo (MCMC) methods (based on simulations and so more time and computing resources consuming) could be used, but many studies show that both perform similarly in a wide range of situations [21, 23].

The difference between the observed deaths and those projected for the year 2020 found by our models was 72,134 deaths, and that could be considered as the global effect of COVID-19. Currently, there is no accurate estimate of the overall impact of COVID-19 during 2020 in Spain. According to the Surveillance System for Mortality Monitorization (MoMo), three periods of excess mortality could be identified during 2020 (March 3 to May 9, July 20 to August 29, and September 1 to December 25). The total observed mortality excess during this three periods accounts for 70,470 deaths (52.9% male and 47.1% female, and 82.8% older than 74 years) [24]. The Carlos III Health Institute (ISCIII) publishes the official figures of deaths from COVID-19, considering as such only those with a positive PCR test or similar, giving a total for the entire year 2020 of 53,569 deaths (54.6% men and 45.4% women, 85.7% of the total older than 70 years) [25]. The INE has published a press release reporting that during the five first months of 2020 (the first and deadliest wave of the epidemic) the death toll attributable to COVID-19 (directly or because this disease acted as comorbidity) was 45,684 (51.5% men, 48.9% women, 87.1% older than 70 years) [26], but the assessment for the second half of the year has yet to be made. All these organizations focus on estimating the direct mortality due to COVID-19, but do not intend in any way to evaluate the global impact considering either indirect deaths or the effects of lockdown period.

All evaluations carried out by the organizations above cited agree on the fact that the effect on mortality has been greater in men and in the older age group. It has also been observed in European countries that the effect of sex on the number of deceased was higher in men that in women (relative risk = 1.60; CI 1.53–1.68) [27]. In another study with data from the first quarter 2020 in Wuhan [28], the authors observed a significantly higher mortality in men compared to women (odd ratio = 3.4; 95% CI 1.2–9.1).

The excess mortality estimates provided by our model, which includes the balance between the different components of excess mortality during the full year 2020, are consistent, albeit different, with those calculated by INE, ISCIII, and MoMo. While INE and ISCIII only show the number of deaths directly attributable to COVID19 (the INE only during the period covering the first wave of the pandemic), MoMo calculates deaths from all causes, but only considering the estimated periods of excess mortality.

Always taking as reference the projected mortality rates for 2020, Madrid was the Autonomous Community that showed the highest increase in the mortality rate, followed by the neighboring community of Castilla-La Mancha. This spread seems to be related to mobility between them in the period prior to lockdown. Similarly, Castilla y León, also adjacent to Madrid, experienced one of the largest increases in mortality. Madrid, Castilla y León and Castilla La Mancha form the central plateau of the Iberian Peninsula, and a very high percentage of Madrid’s population has their roots and strong familiar relationships in those adjacent communities. Madrid was also the European city where the highest excess mortality was recorded during the pandemic, comparing deaths from all causes in Europe in the first half of the year according to the UK Office for National Statistics [29]. In a study on the impact of COVID-19 in metropolitan counties in the USA, it was found that larger metropolitan areas (measured in terms of population) presented higher mortality rates [30]. However, in the present study the effect of territorial contiguity was more influential than the size of the population, since in large cities such as Valencia (Valencian Community) or Seville (Andalusia), the impact of mortality was lower than in cities and towns of Castilla la Mancha adjacent to Madrid.

Catalonia also showed an increase in mortality rates in all age groups, which could be partly attributed to the high occupation rates in the railway and air corridors between Madrid and Barcelona. Catalonia presented the highest daily percentage increase in mortality in Spain (up to 33.96% of daily deaths) in the first 50 days after lockdown [5]. However, in those communities away from Madrid (Galicia, Murcia, Andalusia, and the Balearic, and Canary Islands), mortality rates did not show statistically significant variations in any of the age groups. This could be explained by the fact that when the lockdown began, the number of cases that had reached these communities, especially the island communities, was not large enough to cause an alteration in mortality rates. Other authors reached this same conclusion, based on the low mortality rates observed in the two Spanish autonomous cities in North Africa during lockdown [5]. The lockdown itself could act as a protective factor of mortality for other infectious diseases and thus compensate for the deaths caused by COVID-19. Something similar happened in Italy, where the impact of COVID-19 was much lower in the islands of Sardinia and Sicily [3]. Also, the fact that the mortality rate in Denmark did not increase during the COVID-19 pandemic compared to the mortality rates in the same period during 2015–2019 has been attributed to the protective effect of lockdown measures [31].

## Conclusions

The excess of deaths in Spain in 2020 in relation to projected deaths for that period using the mortality data of the 2008–2019 sequence was 72,146 people, but their spatial distribution was very uneven. In central Spain, the greatest increase in mortality was detected in the adjacent communities of Madrid and Castilla La Mancha. On the contrary, the smallest increments were detected in autonomous communities more distant from Madrid, including the islands, with the only exception of Catalonia. All four models showed good fits (see Fig. 3) and have proved to be a useful tool for estimating the impact of COVID-19 on mortality in Spain in 2020, making it possible to assess how it affected different age groups while accounting for effects of sex, spatial variation between regions and time trend over the last few years.

## Data Availability

Data for this paper are publically available at INE (National Statistics Institute of Spain), in https://www.ine.es/jaxiT3/Tabla.htm?t=10262&L=0, https://www.ine.es/dyngs/INEbase/es/operacion.htm?c=Estadistica_C&cid=1254736177008&idp=1254735573002&menu=resultados#!tabs-1254736195450 and https://www.ine.es/experimental/defunciones/experimental_defunciones.htm. Data and code for fitting the models can also be found at the github site of one of the authors https://github.com/angeloSdP/COVID-19-outburst-Spain.

## Abbreviations

CI: Credibility interval
INE: Instituto Nacional de Estadística (*Spanish National Institute of Statistics*)
ISCIII: Instituto de Salud Carlos III (*Carlos III Health Institute*)
INLA: Integrated Nested Laplace Approximation
MCMC: Markov chain Monte Carlo
MoMo: Sistema de Monitorización de la Mortalidad (*Mortality Monitorization System)*
SMR: Standardized mortality ratio
